# Association of Blood Lead Level with Neurological Features in 972 Children Affected by an Acute Severe Lead Poisoning Outbreak in Zamfara State, Northern Nigeria

**DOI:** 10.1371/journal.pone.0093716

**Published:** 2014-04-16

**Authors:** Jane Greig, Natalie Thurtle, Lauren Cooney, Cono Ariti, Abdulkadir Ola Ahmed, Teshome Ashagre, Anthony Ayela, Kingsley Chukwumalu, Alison Criado-Perez, Camilo Gómez-Restrepo, Caitlin Meredith, Antonio Neri, Darryl Stellmach, Nasir Sani-Gwarzo, Abdulsalami Nasidi, Leslie Shanks, Paul I. Dargan

**Affiliations:** 1 Manson Unit, Médecins Sans Frontières, London, United Kingdom; 2 Public Health Department, Médecins Sans Frontières, Amsterdam, The Netherlands; 3 Medical Statistics Department, London School of Hygiene and Tropical Medicine, London, United Kingdom; 4 Nigeria Mission, Médecins Sans Frontières, Sokoto, Nigeria; 5 Centers for Disease Control and Prevention, National Center for Environmental Health, Atlanta, Georgia, United States of America; 6 Institute of Social and Cultural Anthropology, University of Oxford, Oxford, United Kingdom; 7 Department of Public Health, Federal Ministry of Health, Abuja, Nigeria; 8 Nigeria Centre for Disease Control, Abuja, Nigeria; 9 Guys and St. Thomas' NHS Foundation Trust and King's College London, London, United Kingdom; University of Cincinnati, United States of America

## Abstract

**Background:**

In 2010, Médecins Sans Frontières (MSF) investigated reports of high mortality in young children in Zamfara State, Nigeria, leading to confirmation of villages with widespread acute severe lead poisoning. In a retrospective analysis, we aimed to determine venous blood lead level (VBLL) thresholds and risk factors for encephalopathy using MSF programmatic data from the first year of the outbreak response.

**Methods and Findings:**

We included children aged ≤5 years with VBLL ≥45 µg/dL before any chelation and recorded neurological status. Odds ratios (OR) for neurological features were estimated; the final model was adjusted for age and baseline VBLL, using random effects for village of residence. 972 children met inclusion criteria: 885 (91%) had no neurological features; 34 (4%) had severe features; 47 (5%) had reported recent seizures; and six (1%) had other neurological abnormalities. The geometric mean VBLLs for all groups with neurological features were >100 µg/dL vs 65.9 µg/dL for those without neurological features. The adjusted OR for neurological features increased with increasing VBLL: from 2.75, 95%CI 1.27–5.98 (80–99.9 µg/dL) to 22.95, 95%CI 10.54–49.96 (≥120 µg/dL). Neurological features were associated with younger age (OR 4.77 [95% CI 2.50–9.11] for 1–<2 years and 2.69 [95%CI 1.15–6.26] for 2–<3 years, both vs 3–5 years). Severe neurological features were seen at VBLL <105 µg/dL only in those with malaria.

**Interpretation:**

Increasing VBLL (from ≥80 µg/dL) and age 1–<3 years were strongly associated with neurological features; in those tested for malaria, a positive test was also strongly associated. These factors will help clinicians managing children with lead poisoning in prioritising therapy and developing chelation protocols.

## Introduction

Lead poisoning is not a new phenomenon. Though debate continues as to whether it was described by Hippocrates [1], and the hypothesis that it contributed to the fall of the Roman Empire remains in dispute [2], [3], humans have been exposed to lead as a toxicant since at least the start of industrialisation. It continues to be a significant cause of morbidity. In 2004, lead poisoning accounted for about 0.6% of the global burden of disease and 9 million disability-adjusted-life-years [4]. Lead affects mechanisms as diverse as energy metabolism, apoptosis, cell adhesion, intercellular and intracellular signalling, protein maturation, and genetic regulation [4]. As a result, lead poisoning causes a continuum of sub-clinical and clinical features including hypertension [5], nephropathy [6], infertility [7], anaemia, behavioural changes including violence [8], and decreased IQ (intelligence quotient) [9]; as well as severe manifestations such as acute encephalopathy and death [10], [11], [12].

In resource-rich nations, deaths from lead encephalopathy are a largely historical phenomenon. Hundreds of children died from lead poisoning in the USA in the first half of the 20^th^ Century when lead use was widespread [13]. Lead-related deaths in US cities in the 1950s and 1960s were primarily related to lead paint ingestion [11], [12]. One Baltimore hospital reported 36 cases of severe lead encephalopathy between 1954 and 1956 [14], while 38 cases were reported in Chicago between 1959 and 1963 [15] - examples of a broader problem that reported figures likely grossly underestimate. The last recorded death from lead encephalopathy in the USA was in 2006 [16], preceded by one in 2000 that was itself the first since 1990 [17]. Reports of lead-related deaths are uncommon in resource-poor settings, and mostly detected though outbreaks, such as the 18 deaths in Senegal linked to lead acid battery recycling in 2007 [18].

In March 2010, a Médecins Sans Frontières (MSF) disease surveillance team in Zamfara State, northern Nigeria, was contacted by leaders and health staff of a local village with reports of high mortality in young children following an unknown illness. MSF was invited to assist in investigating these reports by the state Ministry of Health. MSF carried out an initial assessment and rapidly assembled a dedicated response team and 24-hour care in the village clinic. Children presented with sudden onset of abdominal pain and/or vomiting, intractable seizures with or without fever, then sometimes rapid progression to death. Symptoms were unresponsive to initial treatment by the MSF team for common endemic diseases such as malaria and meningitis, and anti-convulsants had little effect. Over 2 months until 17 May 2010, nearly 300 children aged ≤5 years presented in four villages with these symptoms with a mortality of 48%. There were anecdotal reports of a recent increase in small-scale ore processing with dry-milling to extract gold. An outbreak of severe lead poisoning was confirmed [19], [20], [21]. Initially, seven rural villages were identified as extremely lead contaminated due to dust dispersed by artisanal mining activities within the villages, with an estimated 3000 children aged ≤5 years at risk of lead poisoning. Identification activities included active case finding by MSF and investigations by State Ministry of Health with Nigeria Centre for Disease Control and US Centers for Disease Control and Prevention (CDC, USA), and environmental testing by TerraGraphics Environmental Engineering and CDC, USA [22]. The true scale of contamination in the region remains unclear [23]. Extensive environmental remediation has been undertaken [20]. Health promotion has focused on removing the lead exposure harming children by relocating ore processing activities and minimising further lead contamination of the villages.

The focus of the MSF emergency medical response was clinical lead surveillance and chelation therapy. All children aged ≤5 years from the seven villages where remediation was taking place were offered screening by MSF as their villages were remediated. Treatment in the absence of environmental remediation has limited impact, so screening in unremediated villages was considered futile. Children with venous BLL (VBLL) ≥45 µg/dL (the MSF protocol and CDC recommended chelation threshold) from remediated villages [24] were offered chelation therapy, the outcomes of which will be reported separately. Children from unremediated villages presenting to an MSF facility with signs of encephalopathy and therefore at immediate risk of death were tested and treated with chelation therapy if required, and discharged to an uncontaminated location when possible.

Although there has been some characterisation of the clinical pattern of lead toxicity [4], [10], [25] and the VBLL threshold for encephalopathy is often stated to be in the range of 70–100 µg/dL [4], [26], there are limited data on the VBLL threshold above which life-threatening effects including encephalopathy are likely to occur in children [10], [25], [27], [28]. In this paper we describe nearly 1000 lead exposed children with VBLL ≥45 µg/dL, and the clinical and demographic characteristics associated with neurological features using basic clinical examination. This is the largest reported retrospective analysis of data from children ≤5 years with VBLL in this range.

## Methods

For the period June 2010 to the end of June 2011, we included all children aged ≤5 years with a first-ever VBLL ≥45 µg/dL recorded before chelation therapy and whose neurological status was recorded within 7 days of this VBLL. Screening and treatment were provided by MSF. Children were identified via an MSF door-to-door census that detailed children aged ≤5 years living in each residential compound. Screening for enrolment to the MSF chelation programme included a brief clinical history and examination, with particular attention to neurological status. Neurological assessment included history of seizures, change in behaviour, delay or loss of developmental milestones, peripheral neuropathies, gait, assessment of reflexes, and level of consciousness (alert/voice/pain/unresponsive [AVPU] assessment scale [29]). Detailed demographic and clinical data were recorded on standardised forms by medical staff. Only key data were entered into an electronic database, including enrolment data: AVPU, neurological manifestations (summarised as none, present [not severe], severe), recent seizure history (yes/no). In addition, key inpatient data were entered: seizures during hospitalisation (yes/no; if yes, change during admission) and other neurological symptoms during hospitalisation (none, present [not severe], severe; if present, change during admission). Neurological status for this analysis was described by a composite measure of neurological signs or symptoms and AVPU as severe neurological features (1), presumptive seizures (2), mild neurological features (3), or no neurological features identified (4) (Table 1), with categories 1–3 combined as “any neurological features”.

**Table 1:** Neurological status categories and definitions.

	Neurological status category	Definition
1	Severe neurological features:	Seizures witnessed by medical staff; and/or altered consciousness (an AVPU of V or P or U).
2	Presumptive seizures:	A guardian's report of recent (within the past few days) seizure activity and an AVPU of A on presentation; but no seizures witnessed by medical staff.
3	Mild neurological features:	Any neurological signs or symptoms noted by medical staff but without reported history or witnessed seizure; and an AVPU of A on presentation.*
4	No neurological features identified:	No significant neurological signs or symptoms identified on brief clinical examination by the initial treating doctor; and no history of recent seizures.

*Noted abnormalities were hypo-reflexia, inconsolable crying, agitation, and decreased mobility.

“Any neurological features”  =  categories 1+2+3.

VBLL was measured using the Lead Care II point-of-care analyser (Magellan Biosciences, Chelmsford Massachusetts), using manufacturer-recommended protocols, with samples testing above the upper limit of 65 µg/dL retested using a dilution method developed with CDC and described elsewhere [30]. Regular quality control for VBLL was provided by CDC, USA, using inductively-coupled plasma mass spectrometry (ICPMS): point-of-care values of 120 clinical samples (13% diluted in project from >65 µg/dL) were on average 4.0 µg/dL lower than ICPMS (limits of agreement [31] −19.7 µg/dL to +11.7 µg/dL). VBLL results were categorised: 45–64, 65–79, 80–99, 100–119, 120–199, ≥200 µg/dL. Haemoglobin was measured on all venous samples by the HemoCue Hb 301 point-of-care testing system (HemoCue, Angelholm, Sweden) prior to 22^nd^ November 2010, and by the Sysmex Automated Hematology Analyzer, KX-21N (Sysmex, Hyogo, Japan) after this date. Venous samples were also used to assess alanine transaminase (ALT) (HumaLyser 2000 [Human, Wiesbaden, Germany]). Where symptoms such as fever were suggestive of malaria, a malaria rapid diagnostic test (RDT) was performed (HRP-2 tests as the endemic cases and seasonal outbreaks are almost exclusively due to *Plasmodium falciparum*). Prevalence levels in children between the ages of 6 months to 59 months in the area are 48.2% [32]. Children with positive test results were immediately treated with artemisinin-based combination therapy (ACT) for uncomplicated malaria and artemether for severe malaria.

Haematological and biochemical parameters were categorised [33]: haemoglobin (g/dL) low (<10 if <2 years old, <11 if 2–5 years), high (>13 if <2 years, >14 if 2–5 years), or normal; ALT (U/L) normal (0–42), mildly elevated (>42–100), moderately elevated (>100–1000), or severely elevated (>1000). MUAC (mid-upper arm circumference) for nutritional status was recorded as green (≥135 mm), yellow (≥125 to <135 mm), orange (≥110 to <125 mm; moderately malnourished) or red (<110 mm; severely malnourished) for children aged ≥6 months, or not applicable for infants <6 months. Age at time of VBLL was grouped as 0–<6 months, 6–<12 months, 1–<2 years, 2–<3 years, 3–5 years, based on USA Environmental Protection Agency guidance regarding behavioural and physiological development stages for environmental assessment [34].

Selected clinical and laboratory data were routinely entered into an electronic database specifically designed (by JG) to support patient care and programme management. Data were analysed using STATA 10.1 (StataCorp, Texas, USA). Baseline characteristics were described as counts and percentages of patients in each category and compared with chi-squared or Fisher's exact tests unless a high proportion of data was missing. VBLL by neurological status category was calculated as geometric means with 95% confidence intervals (95% CI) due to non-normally distributed data, accommodating clustering by village of residence by using robust standard errors. Log VBLL values by neurological status categories were compared by Analysis of Variance with Scheffe test between groups. Odds ratios (OR) for any neurological features (compared to none) were estimated for the following variables based on review of descriptive data, completeness and plausibility, with random effects (for village of residence as a variable potentially incorporating various unmeasured factors such as level of environmental contamination): gender; age at time of VBLL; baseline VBLL category; nutritional status (MUAC); and haemoglobin. A multivariable multi-level logistic regression random effects model to estimate adjusted OR of the outcome of any neurological symptoms included factors significant at p<0.10 in the unadjusted analysis or with plausible epidemiological or biological association. Backward selection was used to choose prognostic variables for the final model, discarding those no longer associated (p>0.10) with the outcome after adjustment for other variables. P-values were calculated for the strength of association of each variable with the outcome using Wald tests, and the VBLL categorical variable assessed as continuous to test for trend. Interaction was not plausible with the retained variables. The sensitivity of the model was assessed for severity of neurological features. The role of malaria was assessed in patients who had been tested for malaria due to symptoms (33%), adjusting for age and VBLL.

### Ethics statement

This study met the standards set by the independent MSF Ethics Review Board for retrospective analyses of routinely collected programmatic data [35], here being data collected to facilitate life-saving clinical care. These standards include, but are not limited to, assurances of confidentiality, involvement of local partners and minimal harm to patients. Coded identification numbers were used and personal identifiers and all unnecessary data were removed from the dataset. Review of anonymous routinely collected programmatic data does not constitute research under the National Health Research Ethics Committee of Nigeria guidelines. CDC USA staff involvement did not require CDC Human Subject Review as CDC personnel were not involved in treating patients.

## Results

### Patient characteristics

**Figure 1:**
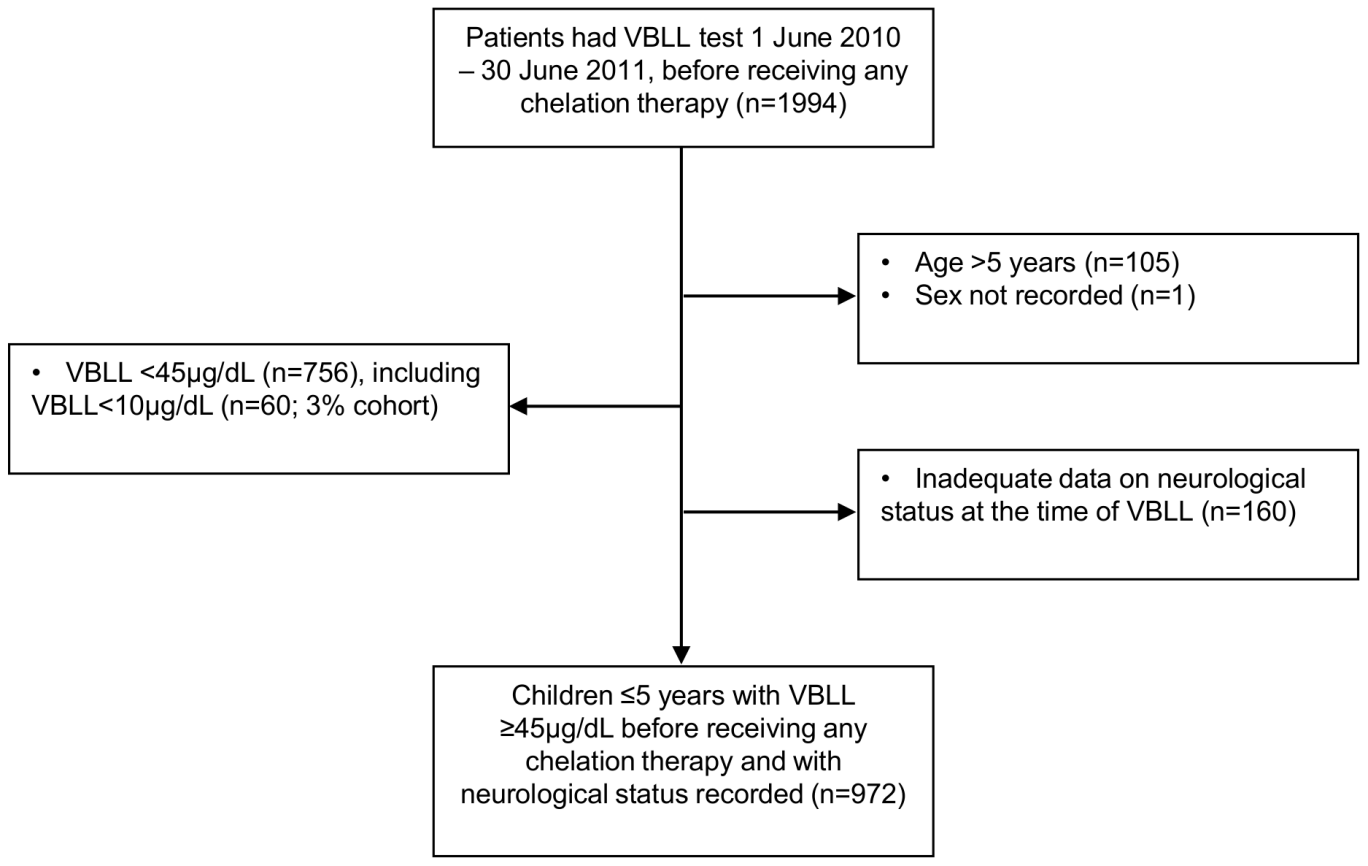
Inclusion and exclusion criteria. VBLL = venous blood lead level.

Between 1 June 2010 and 30 June 2011 approximately 95% of children in the seven remediated villages were screened; 972 children met the inclusion criteria (Figure 1). Among another 160 children meeting age, VBLL and date criteria, but with inadequate record of neurological status in their clinical file, geometric mean VBLL was lower than for those with recorded neurological status (65.3 µg/dL [95%CI 61.5–69.4] vs 79.4 [95%CI 62.6–100.7]; p = 0.010), however there was no difference in the proportion female (46.9% vs 49.2%; p = 0.59), or the age distribution (p = 0.87). Among the 972 children included, 340 (35%) had a VBLL ≥80 µg/dL. The maximum VBLL recorded was 708 µg/dL. 73 (8%) children had a VBLL 120–199 µg/dL and 27 (3%) had VBLL ≥200 µg/dL (Table 2). Proportionally more children aged 1–<2years had VBLL ≥120 µg/dL than in the other age groups (25% [39/159] vs 7% for all other ages combined [61/813; all other age groups were each separately <13%]; p<0.001) (Table 2). 14 children had died by 30 June 2011; lead poisoning was likely a primary cause in five of these deaths as these children had recent high VBLL (104–460 µg/dL) and symptoms consistent with lead encephalopathy, with no other obvious cause of death.

**Table 2:** First-ever pre-chelation VBLL test (in categories, µg/dL) by age group (number and % of row).

	45–64.9	65–79.9	80–99.9	100–119.9	120–199.9	200+	Total	
0–<6 months	33 (65%)	5 (10%)	3 (6%)	5 (10%)	4 (8%)	1 (2%)	**51**	5%
6–<12 months	52 (51%)	5 (5%)	20 (20%)	14 (14%)	7 (7%)	4 (4%)	**102**	10%
1–<2 years	75 (47%)	4 (3%)	23 (14%)	18 (11%)	22 (14%)	17 (11%)	**159**	16%
2–<3 years	53 (54%)	8 (8%)	14 (14%)	11 (11%)	11 (11%)	1 (1%)	**98**	10%
3–5 years	363 (65%)	34 (6%)	88 (16%)	44 (8%)	29 (5%)	4 (1%)	**562**	58%
**Total**	**576 (59%)**	**56 (6%)**	**148 (15%)**	**92 (9%)**	**73 (8%)**	**27 (3%)**	**972**	

VBLL = venous blood lead level.

**Figure 2:**
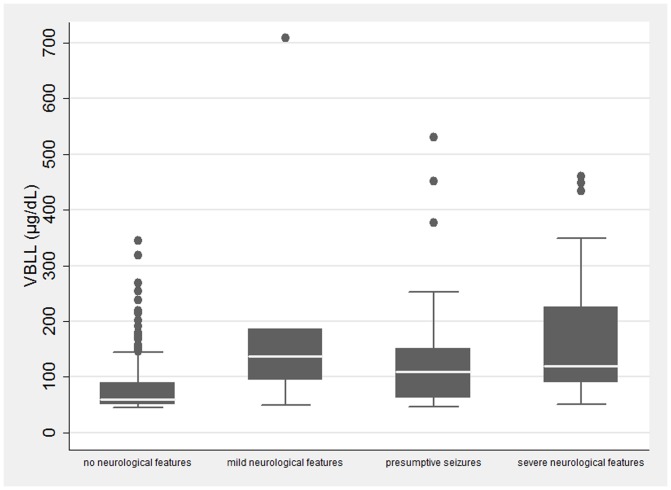
Distribution of VBLL by neurological status in children ≤5 years, with first-ever pre-chelation VBLL ≥45 µg/dL. VBLL tested from 1 June 2010 to 30 June 2011. VBLL = venous blood lead level.

Most (885 [91%]) children had no neurological features, 34 (4%) had seizures witnessed by medical staff and/or reduced level of consciousness, 47 (5%) had a guardian-reported history of recent seizures but no altered consciousness on presentation, and six (1%) displayed some other signs of neurological abnormality (Table 3). The geometric mean VBLL for all groups with neurological features was >100 µg/dL (157.6 µg/dL for mild neurological features, 106.9 µg/dL for presumptive seizures and 170.1 µg/dL for severe neurological features) compared with 65.9 µg/dL for those without neurological features (Table 3; Figure 2). The range of VBLL in each group was wide, such that children with no neurological features at first presentation had VBLLs up to 345 µg/dL, and children with neurological features had VBLL as low as 47 µg/dL (Table 3); only children with concurrent malaria had severe neurological features below VBLL 105 µg/dL.

**Table 3:** Characteristics by neurological status in children ≤5 years, with first-ever pre-chelation VBLL ≥45 µg/dL from 1 June 2010 to 30 June 2011.

	No neurological features (n = 885)	Mild neurological features (n = 6)	Presumptive seizures (n = 47)	Severe neurological features (n = 34)	p-value†	All (n = 972)
Geometric mean VBLL (95% CI) (µg/dL)	65.9 (59.7–72.7)	157.6 (52.1–476.3)	106.9 (76.1–150.3)	170.1 (112.0–258.2)	<0.001	79.4 (62.6–100.7)
VBLL range (µg/dL)	45.0–345.1	49.2–708.0	46.9–531.0	50.6–459.9		45.0–708.0
VBLL ≥80 µg/dL, n (%)	276 (31%)	5 (83%)	32 (68%)	27 (79%)	<0.001	340 (35%)
Sex male, n (%)	450 (51%)	2 (33%)	27 (57%)	15 (44%)	0.56	494 (51%)
Age category, n (%)					<0.001	
0–<6 months	48 (5%)	0	2 (4%)	1 (3%)		51 (5%)
6–<12 months	92 (10%)	1 (17%)	5 (11%)	4 (12%)		102 (10%)
1–<2 years	119 (13%)	3 (50%)	17 (37%)	20 (59%)		159 (16%)
2–<3 years	87 (10%)	1 (17%)	5 (11%)	5 (15%)		98 (10%)
3–5 years	539 (61%)	1 (17%)	18 (38%)	4 (12%)		562 (58%)
Malaria RDT, n (%)						
Symptomatic; RDT negative	80 (9%)	1 (17%)	4 (9%)	7 (9%)		92 (9%)
Symptomatic; RDT positive	177 (20%)	4 (67%)	26 (55%)	26 (76%)		233 (24%)
Asymptomatic/missing*	628 (71%)	1 (17%)	17 (36%)	1 (3%)		647 (67%)
Nutritional status (MUAC), n (%)					0.045	
Red (<110 mm)	8 (1%)	0	2 (4%)	1 (3%)		11 (1%)
Orange (≥110 <125 mm)	23 (3%)	0	5 (10%)	1 (3%)		29 (3%)
Yellow (≥125 <135 mm)	56 (6%)	1 (17%)	3 (6%)	5 (15%)		65 (7%)
Green (≥135 mm)	613 (69%)	3 (05%)	27 (57%)	18 (53%)		661 (68%)
Not applicable (<6 m old)	48 (5%)	0	2 (4%)	1 (3%)		51 (5%)
Missing (≥6 m old)	137 (15%)	2 (33%)	8 (17%)	8 (24%)		155 (16%)
Laboratory tests at time of VBLL, n (%)						
Normal haemoglobin (g/dL)	213 (24%)	0	13 (28%)	3 (9%)	0.050	229 (24%)
Low (<10 if <2 y; <11 if 2–5 y)	664 (75%)	6 (100%)	33 (70%)	30 (88%)		733 (75%)
High (>13 if <2 y; >14 if 2–5 y)	2 (0%)	0	1 (2%)	1 (3%)		4 (0%)
Missing	6 (1%)	0	0	0		6 (1%)
Normal ALT, n (%)	604 (68%)	1 (17%)	26 (55%)	23 (68%)		654 (67%)
Mildly elevated (>42–100 U/L)	73 (8%)	0	2 (4%)	2 (6%)		77 (8%)
Mod. elevated (>100–1000 U/L)	11 (1%)	0	0	1 (3%)		12 (1%)
Severely elevated (>1000 U/L)	0	0	0	0		0
Missing	197 (22%)	5 (83%)	19 (8%)	8 (24%)		229 (24%)

*Malaria test performed only on symptomatic children, but no result in database not confirmation that asymptomatic.

† p-values only given when <20% data missing. VBLL = venous blood lead level. RDT = rapid diagnostic test. MUAC = mid upper-arm circumference. Mod = moderately. m = months. y = years. ALT = alanine transaminase.

Within age groups, neurological features were most common in 1–<2 year olds (25% vs 6% for other ages; p<0.001). 233 (72%) of 325 children tested for malaria had a positive RDT result. A positive malaria test was more common in children with neurological features than in those without (82% [56/68] vs 69% [177/257], respectively; p = 0.034) (Table 3); 19 children with neurological features did not have a malaria test recorded. A positive malaria test was more common in children with VBLL <80 µg/dL (n = 194 tested for malaria) who had any neurological features (95% [21/22]) compared to those without features (72% [123/172]; p = 0.016), but not significantly different with VBLL ≥80 µg/dL (76% [35/46] with neurological features vs 64% [54/85] without; p = 0.14). Poor nutritional status (malnourished [MUAC red or orange] vs not) was more common in children with neurological features than in those without (14% [9/66] vs 4% [31/700], respectively; p = 0.005). Most (75%) children had low haemoglobin regardless of neurological features; a non-significantly higher proportion of children with no neurological features had normal haemoglobin levels (24% vs 18%; p = 0.23). A high proportion (24%) of children had no ALT results recorded; a low proportion of children had elevated ALT levels (9%), none were severely elevated (Table 3).

### Factors associated with neurological features

In logistic regression (unadjusted OR, random effects for village of residence), presentation with any neurological features was not associated with gender (OR = 1.08, 95%CI 0.68–1.72; p = 0.73), or low haemoglobin (OR = 1.39, 95%CI 0.77–2.50; p = 0.27), but was associated with age (highest for 1–<2 years [OR = 9.42, 95%CI 5.23–16.96; p<0.001] compared with 3–5 years), increasing VBLL (test for trend p<0.001, starting from 80.0–99.9 µg/dL with OR = 3.06, 95%CI 1.43–6.53), and moderate-severe malnutrition (OR = 4.76, 95%CI 2.02–11.31; p<0.001). The final multivariate analysis retained only age and VBLL as strongly associated with any neurological features after adjustment for the other factors and random effects for village (both p<0.001). Children aged 1–<2 years had the highest odds of neurological features compared with children age 3–5 years (adjusted OR 4.77, 95%CI 2.50–9.11); age 2–<3 years was also associated with neurological features (adjusted OR 2.69, 95%CI 1.15–6.26) compared with children age 3–5 years. Increasing VBLL (compared with the reference category of 45–64.9 µg/dL) was strongly associated with increased odds of neurological features (test for trend p<0.001): adjusted OR 2.75, 95%CI 1.27–5.98 (VBLL 80–99.9 µg/dL); 3.84, 95%CI 1.62–9.09 (100–119.9 µg/dL); and 22.95, 95%CI 10.54–49.96 (≥120 µg/dL) (Table 4).

**Table 4:** Final multi-level logistic regression model to assess factors associated with having any neurological features at time of first-ever pre-chelation VBLL.

	Unadjusted OR (95% CI)	Adjusted OR (95% CI)	p-value*
Age at time of VBLL			<0.001
0–<6 months	1.35 (0.36–5.04)	1.66 (0.44–6.31)	
6–<12 months	2.07 (0.93–4.63)	1.46 (0.61–3.51)	
1–<2 years	9.42 (5.23–16.96)	4.77 (2.50–9.11)	
2–<3 years	3.54 (1.59–7.88)	2.69 (1.15–6.26)	
3–5 years	1	1	
VBLL			<0.001
45–64.9	1	1	
65–79.9	1.21 (0.26–5.62)	1.02 (0.21–4.87)	
80–99.9	3.06 (1.43–6.53)	2.75 (1.27–5.98)	
100–119.9	4.87 (2.11–11.22)	3.84 (1.62–9.09)	
120+	38.77 (18.14–82.86)	22.95 (10.54–49.96)	

*P-value from the Wald test of no association of the attribute with the outcome adjusted for the other variables in the model. VBLL = venous blood lead level. OR = odds ratio. (n = 972).

A sensitivity analysis using only the outcome of severe neurological features compared with all other patients gave similar overall results. The association with VBLL was stronger and the trend robust (test for trend p<0.001), but significant only from 100–119.9 µg/dL (adjusted OR 7.35, 95%CI 2.02–26.79; for VBLL≥120 µg/dL adjusted OR 23.96, 95%CI 7.33–78.31). The adjusted OR were also higher for all ages with the largest associations for children aged 1–<3 years (OR>6 for both 1–<2 y and 1–<3 y) compared with children aged 3–5 years.

Severe neurological features in children with a positive malaria RDT result were seen at VBLL as low as 50 µg/dL, while in those with a negative RDT the lowest VBLL with severe neurological features was 105 µg/dL, and in the one child with severe neurological features and no malaria test result the VBLL was 293 µg/dL. In the 325 children with a malaria RDT result, the unadjusted OR of a positive RDT with any neurological features was 2.04 (95% CI 0.96–4.35; p = 0.065). After adjustment for age and VBLL there was evidence of a strong association of malaria with neurological features (adjusted OR = 3.60, 95% CI 1.42–9.09; p = 0.007); after adjustment for malaria, age and VBLL showed a similar pattern of association with neurological features as in the entire cohort, that is, highest adjusted OR in 1–<2 year olds and increasing adjusted OR with increasing VBLL.

## Discussion

The MSF programmatic data collected during the response to the Zamfara lead poisoning disaster presents a cohort size and severity that is unprecedented. While lead poisoning was endemic in resource-rich countries in the early to mid-20^th^ Century, an acute outbreak of this magnitude has not been reported in the literature. Chisolm *et al*. reported 197 children exposed to lead including 41 cases of severe encephalopathy in 1952–1954 [12] and Greengard *et al*. reported 38 severe cases in 1962 and 1963 with blood lead levels in 20 fatalities of 90–825 µg/dL [15]. More recently, the Treatment of Lead-exposed Children (TLC) trial group reported a cohort of 780 patients, although all had VBLL <45 µg/dL [36] and thus are not comparable with the patients in Zamfara. Reports in recent decades with similar numbers of children screened for lead poisoning have been based on general population datasets, with much lower geometric mean VBLLs, generally <10 µg/dL [37], [38]. Media reports of lead poisoning of hundreds of children in China in 2009 [39], [40] indicated comparatively low lead levels, with no detailed reports published of acutely severely intoxicated children. Other recent studies have concerned low-level endemic lead poisoning [41], [42]. In the 972 children included in this analysis, the geometric mean VBLL was 79.4 µg/dL, 35% had VBLL ≥80 µg/dL, and 9% had some clinically identified neurological features consistent with lead poisoning. An additional 696 children (35% of those screened) had first-ever VBLLs of 10–44.9 µg/dL and thus did not require chelation (and are not reported in this analysis), such that the range of exposure of this cohort of children is consistent with the entire clinical and sub-clinical spectrum of lead pathophysiology.

Children with severe neurological features of altered consciousness or seizures witnessed by clinical staff had significantly higher VBLL than those with only a guardian-reported recent history of seizures. Having any neurological feature was strongly associated with increasing initial VBLL, with evidence of an increased OR at a VBLL of 80–99.9 µg/dL, becoming stronger at 100–119.9 µg/dL and larger still from 120 µg/dL. This is consistent with previous ranges given for the threshold above which encephalopathy risk is increased [4], , strengthened by the robust evidence from this large cohort of poisoned children. Clinicians managing patients with lead poisoning should take into consideration the presence of neurological features and, particularly, the potential risk of life-threatening encephalopathy at VBLL above 80 µg/dL, particularly (as discussed in more detail below) in those with concurrent malaria.

Severe neurological features were not seen below VBLL 50 µg/dL and in the absence of a positive malaria test, not below 105 µg/dL. Most children with neurological features at a lower VBLL (<80 µg/dL) also had a positive malaria test. In 325 children with symptoms suggestive of malaria who were tested by RDT, a positive result was independently associated with neurological features in addition to the trend associating increasing VBLL with neurological features. It is challenging to distinguish between features of cerebral malaria and lead encephalopathy clinically, and the presence of both lead and malaria were strongly associated with neurological features. We postulate that it is also possible that haemolysis associated with malaria increases the relative proportion of free plasma lead at a given (whole blood) VBLL, increasing the lead available to cause encephalopathy. The interaction between malaria infection and lead toxicity is still uncertain and is an area for future research.

Of children with no neurological features at the time of initial examination, 31% had VBLL ≥80 µg/dL, comparable to the United States National Academy of Sciences cohort [10]. Clinical signs and symptoms varied among children with similar blood lead levels, confirming that clinical features alone are poor predictors of the severity of lead poisoning and the risk of long-term neurological damage [21]. This variation may be due to a number of factors including duration of exposure and other factors which may influence absorption, storage, and effects of lead in the body such as genetic polymorphisms [43], co-morbidities, and essential trace element deficiency. The limited sensitivity and specificity of the neurological assessments and categorisation may also have contributed to the recorded variation.

A larger proportion of children with neurological features were aged 1–3 years compared with those without neurological features. The greatest proportion of high VBLLs were seen in children aged 1–<2 years, and the strongest association with neurological features was in this age group after adjusting for VBLL. During the initial outbreak investigation (before lead poisoning was confirmed and before chelation therapy began) proportionally more children aged 1–<2 years died amongst children displaying probable or suspected neurological features. We hypothesise that this risk pattern for both high VBLL and stronger association with neurological features may reflect a number of factors: variation in bioavailability of ingested lead; variation in exposure due to behavioural (i.e., hand-mouth) and developmental factors; immaturity of the blood/brain barrier compared with older children and possibly other age-related influences on activities and movement around the contaminated villages and ore processing areas. Conversely, there are several reasons why children younger than 1 year may have been less at risk of raised VBLL and neurological effects: shorter duration of potential exposure by virtue of younger age; poorer mobility and less marked hand-mouth behaviour; and physiological variation in absorption. However, the neurological damage done by lead, beyond fatal lead encephalopathy, is long-term [4], [26] and only continued monitoring will allow us to fully document and understand the long-term sequelae of lead toxicity in this cohort.

Lead poisoning related to industrial activity is an ongoing problem, with a recent outbreak in Senegal secondary to used lead acid battery recycling linked to 18 child deaths and 81 additional cases of poisoning [18]. In the United Nations-Administered Province of Kosovo, camps for internally displaced people were contaminated by industrial lead production, and children born there between 1999 and 2007 had lead levels up to 74 µg/dL [44]; children near a recently closed auto-battery recycling plant in the Dominican Republic had venous blood lead levels up to 130 µg/dL [45]; and concerns continue about widespread exposure of children to lead from industries in China [36], [46]. Other heavy metals such as mercury and cadmium also continue to cause contamination [47] and poisoning associated with industrial development [48], [49]. With increased industrialisation in resource-poor countries and dumping of toxic waste, we expect greater frequency of undetected lower level poisoning and an increased risk of severe environmental toxicological emergencies. Whilst the Zamfara outbreak was at the most severe end of the spectrum, the true extent of lower-level poisoning and the implications for those exposed are unclear.

Limitations to our data arise from the circumstance in which they were collected, namely an emergency humanitarian response to mass mortality and morbidity from lead poisoning. While screening was offered to all children in the seven villages remediated in the first year, tragically, hundreds of children had died before the excess mortality was notified and cause identified in these villages; these children were not included in this analysis. In the first 2 months of the response, 42 children did not have VBLL recorded immediately prior to starting chelation therapy (with an earlier screening BLL ≥65 µg/dL) due to logistical challenges, including three who had had witnessed seizures and three with presumptive seizures; these children were not included due to the likely effect of chelation on any VBLL taken after therapy was started. Blood for VBLLs from these children were taken 1–11 days after commencing chelation therapy; 70% were still ≥80 µg/dL (range 15–241 µg/dL). Thus potentially some of the most severely poisoned children were not included in this analysis. The neurological data collected may have missed subtle neurological abnormalities that would not have been identified with the simple tools used that focused on detecting life-threatening encephalopathy. The VBLLs in the first months may not be precise due to the unanticipated need to determine appropriate dilution methods for the portable testing equipment, and because the Lead Care II will not be as precise as ICPMS. However, quality control showed that Lead Care II values were on average only 4.0 µg/dL lower than those measured using ICPMS. In addition, the multivariable model was assessed for sensitivity to excluding VBLL tests in the first months and the pattern of associations were not substantially altered (data not shown). Children with inadequately recorded neurological status at time of VBLL had a lower geometric mean VBLL, which may indicate that these children were less likely to have exhibited neurological features. A sensitivity analysis of the model including all these patients as having had no neurological features did not alter the patterns of association (data not shown). The date of birth is often inaccurate or unknown in this remote, rural region, therefore most ages in this analysis are approximations, and thus age was categorised. In addition there was a substantial amount of missing data for some important variables such as the malaria RDT result. The fact that only symptomatic children were tested suggests that clinical malaria was present; however, as the RDT may be positive for weeks after treatment, microscopy would be needed to confirm acute infection. There is the potential that the neurological features seen may be related to comorbidities other than malaria or to permanent damage from previous high VBLLs. As with any large dataset, despite standardised forms, continuous use of data for clinical purposes, and regular data cleaning, it is possible that there was some misclassification, but this is considered unlikely to be differential. The strength of this dataset is its unequalled size, severity of lead exposure, and scope of routinely collected clinical data.

## Conclusion

In this large cohort of 972 children exposed to environmental lead contamination, 35% of children ≤5 years with a VBLL requiring chelation therapy (≥45 µg/dL) had VBLLs ≥80 µg/dL. There was evidence that a VBLL ≥80 µg/dL was associated with neurological features and strengthened as VBLL increased, and neurological features were also more likely in children aged 1–<3 years compared to those 3–5 years. Severe neurological features were seen at VBLL <105 µg/dL only if there was a concurrent positive malaria test, and among children tested for malaria, a positive malaria RDT result was strongly associated with neurological features after adjustment for age and VBLL. These results will help clinicians managing children with lead poisoning to determine which individuals are likely to be at greatest risk of developing neurological features and therefore potentially life-threatening encephalopathy. This is particularly important when managing large outbreaks for determining urgency of therapy and to help guide chelation protocols.
